# Exogenous abscisic acid application decreases cadmium accumulation in *Arabidopsis* plants, which is associated with the inhibition of IRT1-mediated cadmium uptake

**DOI:** 10.3389/fpls.2014.00721

**Published:** 2014-12-16

**Authors:** Shi Kai Fan, Xian Zhi Fang, Mei Yan Guan, Yi Quan Ye, Xian Yong Lin, Shao Ting Du, Chong Wei Jin

**Affiliations:** ^1^Ministry of Education Key Laboratory of Environment Remediation and Ecosystem Health, College of Environmental and Resource Science, Zhejiang UniversityHangzhou, China; ^2^College of Environmental Science and Engineering, Zhejiang Gongshang UniversityHangzhou, China

**Keywords:** abscisic acid, cadmium, cadmium translocation, iron, IRT1

## Abstract

Cadmium (Cd) contamination of agricultural soils is an increasingly serious problem. Measures need to be developed to minimize Cd entering the human food chain from contaminated soils. We report here that, under Cd exposure condition, application with low doses of (0.1–0.5 μM) abscisic acid (ABA) clearly inhibited Cd uptake by roots and decreased Cd level in *Arabidopsis* wild-type plants (Col-0). Expression of *IRT1* in roots was also strongly inhibited by ABA treatment. Decrease in Cd uptake and the inhibition of *IRT1* expression were clearly lesser pronounced in an ABA-insensitive double mutant *snrk2.2/2.3* than in the Col-0 in response to ABA application. The ABA-decreased Cd uptake was found to correlate with the ABA-inhibited *IRT1* expression in the roots of Col-0 plants fed two different levels of iron. Furthermore, the Cd uptake of *irt1* mutants was barely affected by ABA application. These results indicated that inhibition of *IRT1* expression is involved in the decrease of Cd uptake in response to exogenous ABA application. Interestingly, ABA application increased the iron level in both Col-0 plants and *irt1* mutants, suggesting that ABA-increased Fe acquisition does not depend on the IRT1 function, but on the contrary, the ABA-mediated inhibition of *IRT1* expression may be due to the elevation of iron level in plants. From our results, we concluded that ABA application might increase iron acquisition, followed by the decrease in Cd uptake by inhibition of IRT1 activity. Thus, for crop production in Cd contaminated soils, developing techniques based on ABA application potentially is a promising approach for reducing Cd accumulation in edible organs in plants.

## Introduction

Cadmium (Cd) is recognized as an important pollutant due to its high toxicity. In the human body, Cd adversely affects kidneys and bones (Ronald, 2000; Pan and Wang, 2012). Cd contamination of agricultural soils because of rapid industrial development and release of agrochemicals into the environment is an increasingly serious problem. The primary route of Cd entry into the human body is through crops grown in Cd-contaminated soil (Meharg et al., 2013). Therefore, in recent decades, scientists have made great efforts to identify strategies for reducing/avoiding Cd accumulation in crops grown in Cd contaminated soils. Many different strategies have been proposed for remediating Cd-contaminated soil to prevent Cd uptake by crops. These include the following: (1) “dig-and-dump” or encapsulation of the contaminated soil (Pulford and Watson, 2003; Khan et al., 2004); (2) chemical immobilization or extraction of Cd (Chen et al., 2000; Muehe et al., 2013); (3) phytoremediation by Cd-hyperaccumulating plants (Marques et al., 2009; Gupta et al., 2013); (4) screening or breeding of crop genotypes with lower Cd accumulation (Liu et al., 2010; Meharg et al., 2013); and (5) proper fertilization for immobilizing Cd in soil or for reducing Cd uptake by roots of crops (Sarwar et al., 2010; Luo et al., 2012; Mao et al., 2014).

Recent studies have showed that the application of plant hormones such as abscisic acid (ABA) might be a promising strategy for reducing Cd accumulation in crops. Hsu and Kao reported that during Cd exposure, the rice (*Oryza sativa* L.) plants pre-treated with ABA showed lesser Cd uptake than by those without ABA pre-treatment (Hsu and Kao, 2003). Similar results have been also observed by Uraguchi et al. (2009). However, in these two reports, extremely high ABA concentrations were used for pre-treatment (5 and 100 μM, respectively). High doses of ABA may have toxic effects on the plants, which may negatively affect the growth and physiological functions of roots. If this was the case, the inhibitory effect of ABA pre-treatment on Cd uptake in the above two studies may be because of the toxic effect of the high dose of ABA rather than the signaling functions of ABA *per se*. As a result, application of high-doses of ABA for decreasing Cd level in plants, also probably leads to the decrease in biomass production. Such a strategy might be not suitable for practical crop production in regions where the food supply is insufficient. Therefore, it is necessary to investigate the effect of low-dose ABA on Cd uptake by plant roots. If low-dose ABA decreases Cd entry into plants, and improves (or at least does not decrease) biomass production, this might be also a practicable strategy for reducing Cd accumulation in crops grown in Cd contaminated soils.

The mechanism of ABA induced decrease in Cd accumulation in plants is also of interest. IRON-REGULATED TRANSPORTER 1 (IRT1) is a divalent plasma membrane cation transporter essential for the uptake of ferrous iron from the soil in non-germinaceous monocots and dicots (Vert et al., 2002; Kobayashi and Nishizawa, 2012). However, IRT1 is a broad-spectrum transporter, and it participates in the absorption of several other divalent cations (Lux et al., 2011; Nishida et al., 2011). Previous studies have shown that, in the presence of Cd, loss in function of IRT1 in *irt1* mutants leads to a marked decrease in Cd levels in plant tissues, indicating that IRT1 is a key transporter responsible for Cd uptake by roots from the growth medium (Vert et al., 2002). Seguela et al. (2008) showed that exogenous application of ABA greatly inhibited *IRT1* expression in *Arabidopsis* roots. On the basis of these reports, we speculated that exogenous ABA application might decrease Cd uptake via inhibition of IRT1.

In this study, wild-type *Arabidopsis* ecotype Col-0 plants and an ABA insensitive mutant *snrk2.2/2.3* were used to investigate the effect of low-dose ABA on Cd uptake in plants. Two IRT1-null mutants, *irt1-1* and *irt1-2*, were also used to clarify the correlation between decrease in Cd uptake and inhibition of IRT1 activity in response to ABA application. Evidence presented in this study indicates that application of low-dose ABA decreases Cd levels in plants, which is associated with the inhibition of IRT1 activity in roots.

## Materials and methods

### Plant material

The Col-0 ecotype of *Arabidopsis* and mutants generated in its background were used in this study, including an ABA-insensitive double mutant *snrk2.2/2.3* (Fujii et al., 2007) and two IRT1-null mutants, *irt1-1* and *irt1-2* (Fukao et al., 2011). The *snrk2.2/2.3* seeds and the *irt1-1* and *irt1-2* seeds were kind gifts from Dr. Jian-Kang Zhu (Purdue University, USA) and Dr. Takafumi Mizuno (Mie University, Japan), respectively.

### Hydroponic culture

Seeds were germinated on a nylon net floating in complete nutrient solution [750 μM NaH_2_PO_4_, 500 μM MgSO_4_, 375 μM K_2_SO_4_, 2.25 mM KNO_3_, 375 μM (NH_4_)_2_SO_4_, 1 mM CaCl_2_, 10 μM H_3_BO_3_, 0.5 μM MnSO_4_, 0.5 μM ZnSO_4_, 0.1 μM CuSO_4_, 0.1 μM (NH_4_)_6_Mo_7_O_24_, and 25 μM Fe-EDTA, pH 5.8]. On day 7, after germination, the seedlings were transferred to sand supplemented with fresh complete nutrient solution. After 10 days, batches of four seedlings were transplanted to 0.4 L pots filled with complete nutrient solution. At 5 weeks of age, the plants were transferred to the following media: complete nutrient solution alone; complete nutrient solution with varying concentrations of ABA (as indicated in the figures); complete nutrient solution with 10 μM CdCl_2_, complete nutrient solution with both 10 μM CdCl_2_ and varying concentrations of ABA. The growth media were renewed daily. The plants were harvested for further analysis after 3 or 7 days of treatments.

For exogenous ABA treatment with different iron concentrations, 5-week-old plants were transferred to 10 μM Cd-added nutrient solutions containing 0.0 or 0.5 μM ABA. The iron concentration in the nutrient solutions was maintained at 25 μM or decreased to 5 μM. The growth mediums in all treatments were renewed daily. The plants were harvested for further analysis after 3 or 7 days of treatments.

### Measurement of biomass and metal concentrations

After harvest, the plants were separated into shoots and roots with scissors. Roots were washed three times with deionized water and blotted dry with a paper towel. The fresh weights of shoots and roots were then recorded. Thereafter, the plant tissues were dried at 80°C for 48 h. The dried samples were wet digested as previously described (Jin et al., 2009). Digestates were diluted by ultrapure water, and the concentrations of Cd and Fe in the digestates were analyzed by Thermo Scientific AAS (iCE 3300).

### Real-time reverse transcription-PCR analysis

The root samples were ground in liquid nitrogen. The total RNA was extracted with TRIzol. The first-strand cDNA was synthesized with the total RNA by PrimeScript reverse transcription (RT) reagent kit (TaKaRa). The mRNA levels of *IRT1* were detected by the SYBR Green RTPCR kit (TaKaRa) with the following pair of gene-specific primers: fw, AAGCTTTGATCACGGTTGG; rev, TTAGGTCCCATGAACTCCG. The RT-PCR analysis was performed with MJ Option™ 2Real-Time PCR System (MJ Research™) with the following cycling conditions: 30 s at 95°C, 40 cycles of 95°C for 5 s, 55°C for 30 s, 72°C for 30 s. A pair of *UBQ10* housekeeping gene primers was used for a control in the PCR: fw: ACCCTAACGGGAAAGACGA; rev, GGAGCCTGAGAACAAGATGAA. Amplification of PCR products was monitored via intercalation of SYBR-Green Relative expression of *IRT1* was calculated according to the equation as described previously (Jin et al., 2009).

### Statistics

All statistical analyses were conducted with SAS software (SAS Institute, Cary, NC). Means were compared by *t* test or Fisher's least significant difference test at *P* < 0.05 in all cases.

## Results

### Effect of ABA application on Cd tolerance

**Figure 1:**
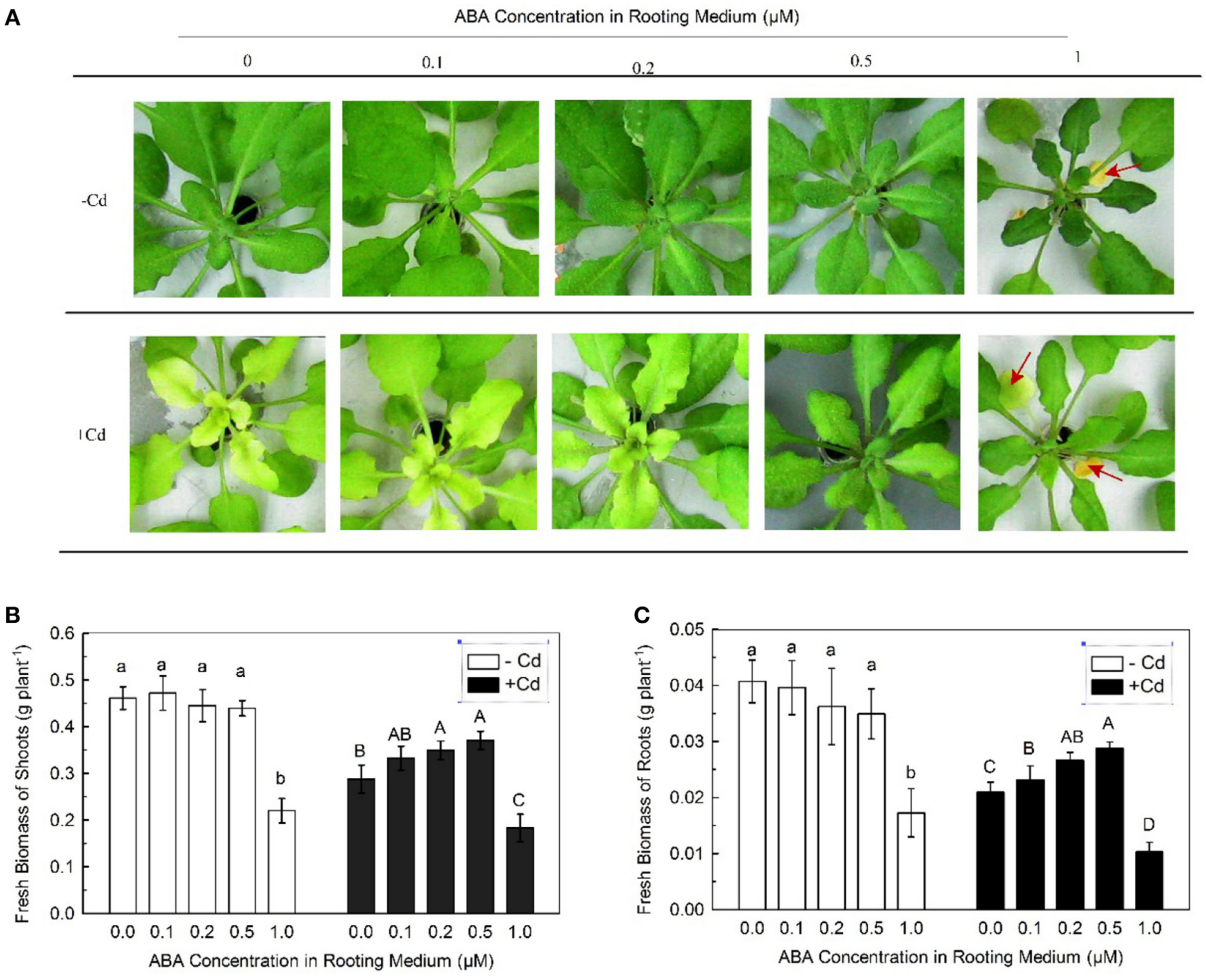
**Effects of varying doses of ABA on Cd tolerance of Col-0 plants**. Plants were grown in Cd-free (−Cd) or 10 μM Cd-added (+Cd) nutrient solution with varying concentrations of ABA added for 7 day. The Fe level in the nutrient solution was 25 μM. **(A)** Leaf chlorosis. **(B)** Shoot biomass. **(C)** Root biomass. Red arrows indicate necrotic leaves. Data are means ± SD (*n* = 8). Different letters represent significantly different values at *P* < 0.05.

Chlorosis of leaves is an apparent symptom of Cd stress to plants (Dalcorso et al., 2008). After 7 days of exposure to 10 μM Cd, the newly formed leaves of Col-0 plants showed severe chlorosis (Figure 1A). Because high-dose ABA may be toxic to plants, a series of low concentrations of ABA (0.1–1 μM) were applied to the plants. The Cd-induced chlorosis was clearly alleviated by the ABA treatment, with increased alleviation when the ABA concentration was increased. At 1 μM ABA concentration, the oldest mature leaves (indicated using red arrows in figure) in both Cd-free and Cd-exposed plants became necrotic and wilted (Figure 1A), indicating that a high dose of ABA is toxic to plants regardless of Cd exposure.

Inhibition of growth is another typical symptom of Cd stress to plants (Jin et al., 2013; Mao et al., 2014). Therefore, the effect of exogenous ABA on Cd-inhibited growth was also investigated. In the absence of Cd, applications of 0.1–0.5 μM ABA showed no significant decrease in shoot and root biomasses. In the presence of Cd, shoot and root biomasses showed improvement with increase in ABA concentrations. However, at 1 μM ABA concentration, both shoot and root biomasses were greatly decreased regardless of Cd exposure (Figures 1B,C), providing additional evidence that high-dose ABA is toxic to plants. These results suggested that application with low-dose ABA alleviates Cd toxicity in plants.

### Effect of ABA application on Cd uptake

**Figure 2:**
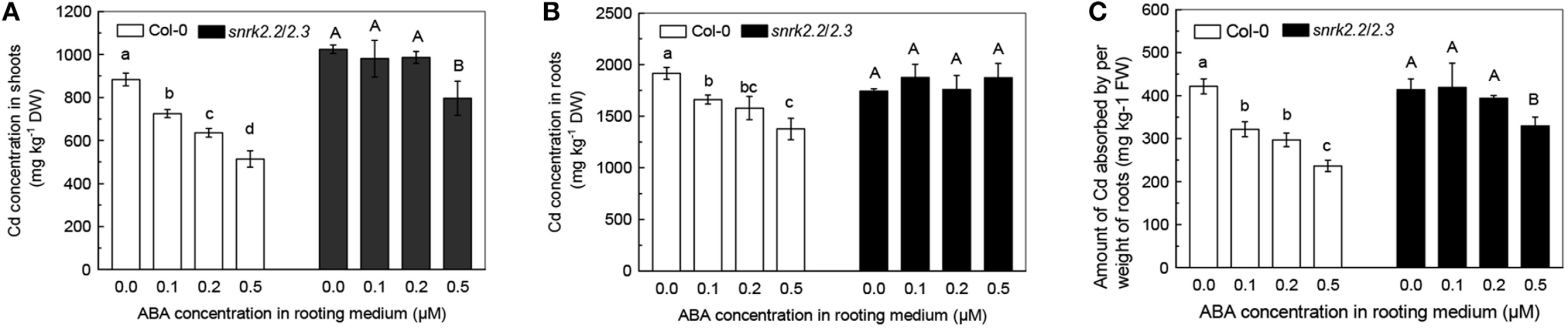
**Effects of varying doses of ABA on Cd levels and Cd uptake in Col-0 and *snrk2.2/2.3* plants**. Plants were grown in a nutrient solution with 10 μM Cd and varying concentrations of ABA added for 7 days. The Fe level in the nutrient solution was 25 μM. **(A)** Cd concentration in shoots. **(B)** Cd concentration in roots. **(C)** Amount of Cd absorbed by per weight of roots (ACAPR). Data are means ±SD (*n* = 5). Different letters represent significantly different values at *P* < 0.05.

The level of a toxic metal accumulation in plants is a crucial factor in determining the toxic effects of the metal on plants (Das et al., 1997). Since 1 μM ABA negatively affected plant growth, we measured Cd levels in the Cd-exposed plants from 0.0 to 0.5 μM ABA treatments. As shown in Figures 2A,B, increase in ABA concentrations significantly decreased the Cd levels in both shoots and roots of Col-0 plants. In the 0.5 μM ABA treatment, the Cd levels in shoots and roots were respectively 40 and 30% less as compared with those in the ABA-free treatment. In addition, the amount of Cd absorbed per weight of roots (ACAPCR) decreased with increase in ABA levels (Figure 2C). These results indicated that application of low-dose ABA inhibits Cd uptake by roots, thus decreasing Cd accumulation in plants.

We also investigated the effect of ABA treatment on Cd levels in ABA insensitive double mutant *SnRK2.2/2.3* in which the SnRK2.2 and SnRK2.3 mediated ABA signaling pathways are null (Fujii et al., 2007). As compared with ABA-free treatment, 0.1 or 0.2 μM ABA application showed no marked effect in both shoots and roots of the mutant plants. Treatment with 0.5 μM ABA also had little effect on Cd level in roots of the mutant plants. These results were in contrast with those obtained for Col-0 plants (Figures 2A,B). In addition, although 0.5 μM ABA application decreased the Cd level in shoots of *snrk2.2/2.3* mutant plants significantly, the decrease was less than that observed in Col-0 plants (Figures 2A,B). The decreases in ACAPCR were also clearly less pronounced in the *snrk2.2/2.3* mutant plants than in the Col-0 plants in response to varying doses of ABA (Figure 2C). These results indicated that inhibition of Cd uptake by treatment with low dose of ABA in wild-type plants should be dependent on the signaling functions of ABA, rather than its toxic effect. Because 0.5 μM ABA showed the greatest inhibition on the Cd uptake of roots, all of the subsequent experiments were performed using 0.5 μM ABA.

### ABA application inhibits *IRT1* expression in roots but increases Fe levels in plants

**Figure 3:**
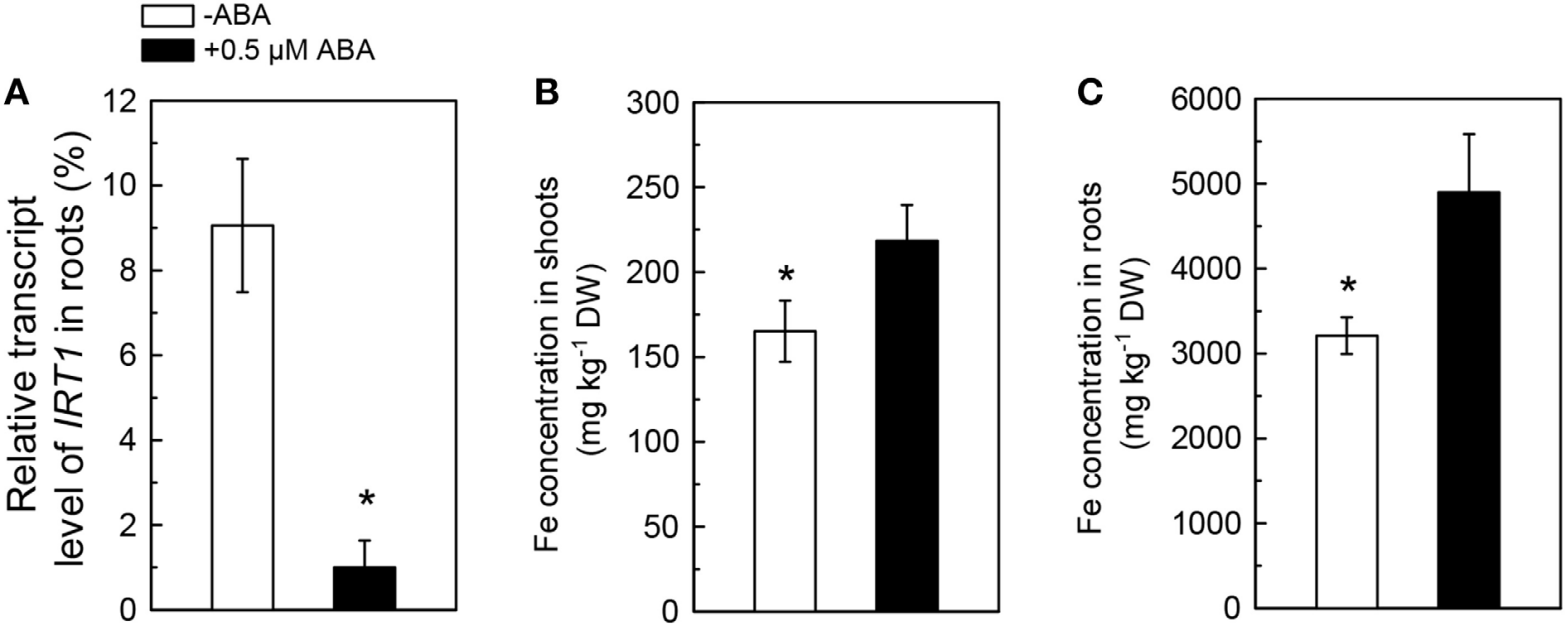
**Effects of ABA on *IRT1* expression and Fe concentration in Col-0 plants**. Plants were grown in 10 μM Cd-added nutrient solution containing 0 or 0.5 μM ABA. The Fe level in the nutrient solution was 25 μM. The expression of ***IRT1*** and Fe concentration were analyzed after 3 and 7 days of treatments, respectively. **(A)** Expression of ***IRT1*** in roots. **(B)** Fe concentration in shoots. **(C)** Fe concentration in roots. Transcript level of *IRT1* was normalized to that of *UBQ10* mRNA (100%). Data are means ± SD (*n* = 5).^*^Significant differences (*P* < 0.05) between ABA-free and ABA-added treatments.

**Figure 4:**
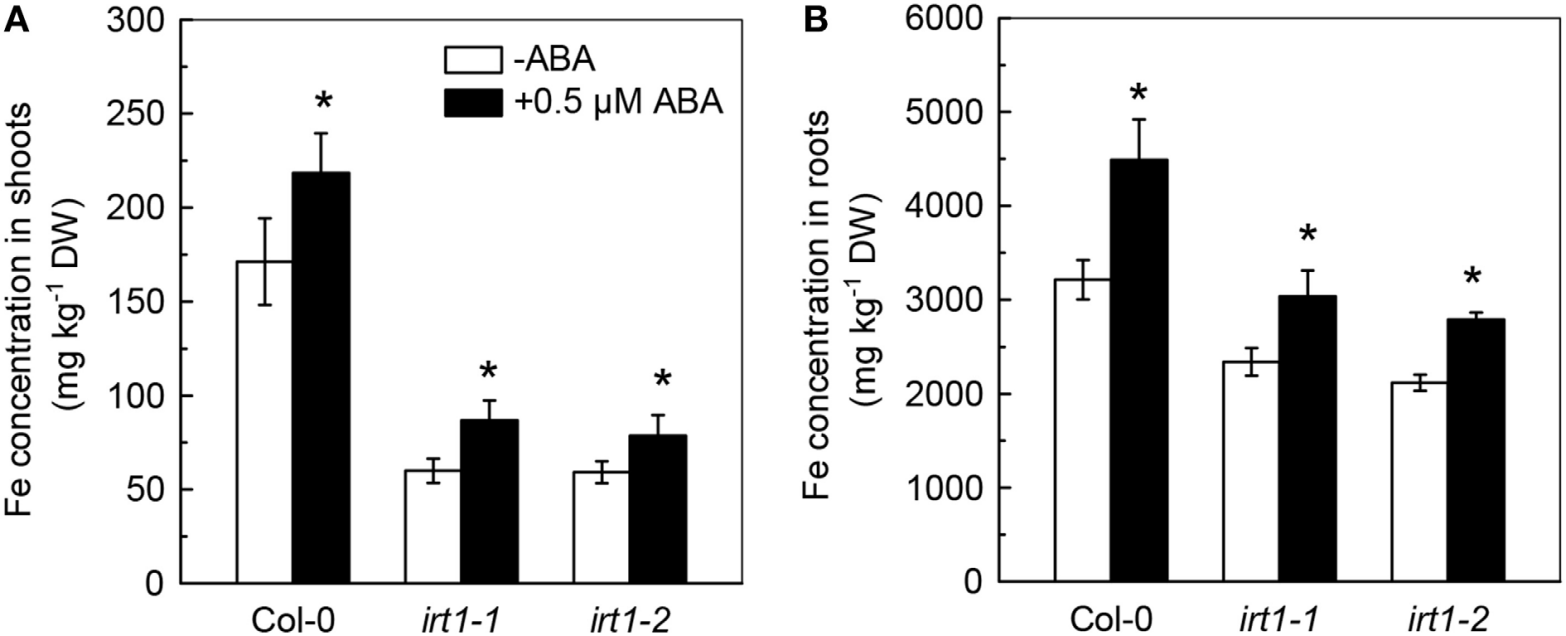
**Effect of ABA on Fe concentration in Col-0 plants and the *irt1-1* and *irt1-2* mutants**. Plants were grown in 10 μM Cd-added nutrient solution containing 0 (−ABA) or 0.5 μM ABA for 7 days. The Fe level in the nutrient solution was 25 μM. **(A)** Fe concentration in shoots. **(B)** Fe concentration in roots. Data are means ± SD (*n* = 5). ^*^Significant differences (*P* < 0.05) between ABA-free and ABA-added treatments.

Because the ABA-decreased Cd uptake was previously assumed to be associated with an inhibition in activity of IRT1, the primary Fe transporter in roots, the effects of ABA application on *IRT1* expression and Fe concentration in Cd-exposed plants were analyzed. As compared with the ABA-free treatment, the 0.5 μM ABA application resulted in about 90% decrease in the transcript level of *IRT1* in roots of Col-0 plants (Figure 3A). However, 0.5 μM ABA application increased Fe levels in both roots and shoots (Figures 3B,C), indicating that ABA-increased Fe uptake in plants might not be associated with IRT1. We then investigated the effect of exogenous ABA on Fe concentration in two IRT1-null mutants, *irt1-1* and *irt1-2*. After 7 days of 0.5 μM ABA treatment, increased Fe levels were observed in shoots and roots of these two Cd-exposed *irt1* mutants (Figures 4A,B). These result verified the above speculation that IRT1 is not involved in the process of ABA increasing Fe uptake.

### Inhibition of IRT1 is responsible for the ABA-inhibited Cd uptake

**Figure 5:**
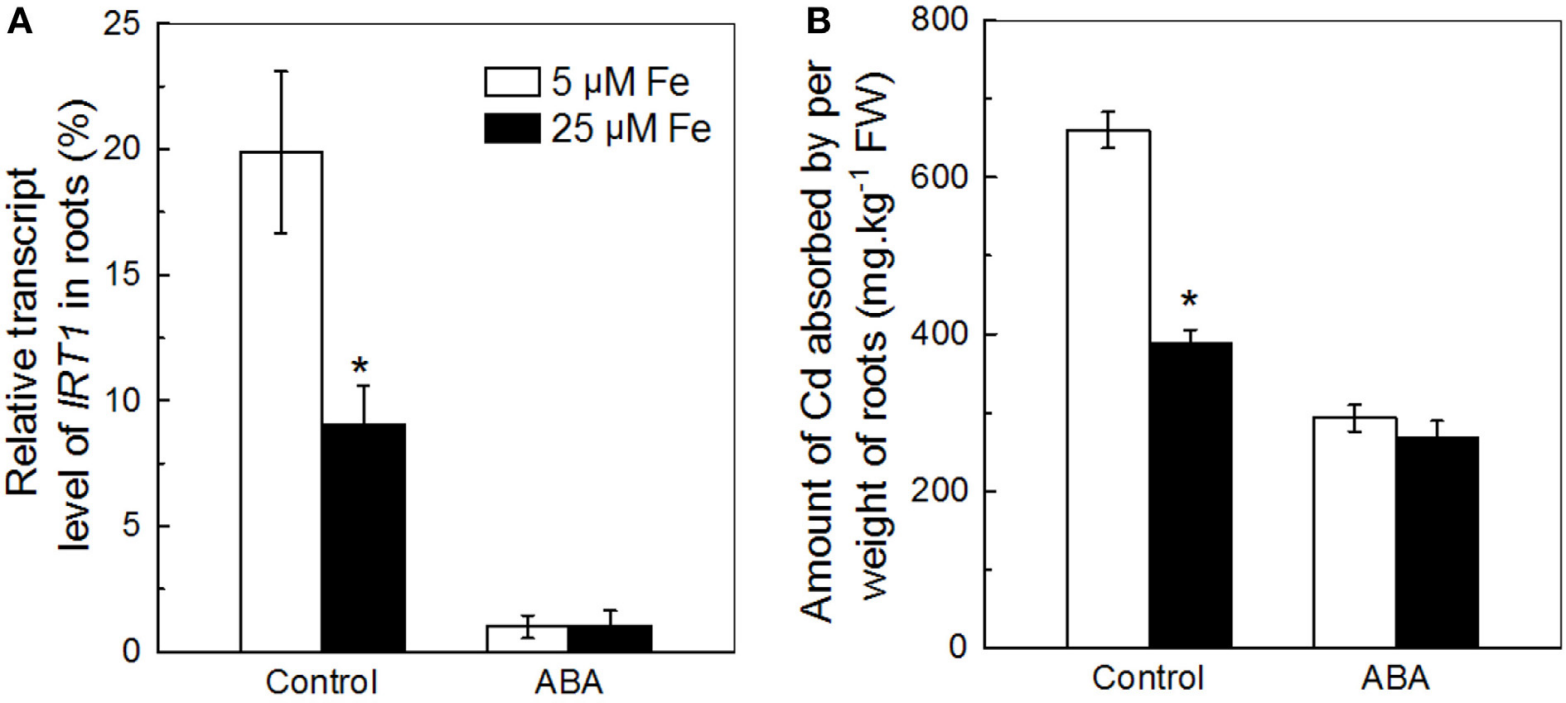
**Effects of ABA on (A) *IRT1* expression and (B) Cd uptake in roots of Col-0 plants supplied with different levels of Fe**. Plants were grown in 10 μM Cd-added nutrient solution containing 0 or 0.5 μM ABA. The Fe level in the nutrient solution was 25 or 5 μM. The expression of ***IRT1*** and the amount of Cd absorbed by per weight of roots were analyzed after 3 and 7 days of treatments, respectively. Data are means ± SD (*n* = 5). ^*^Significant differences (*P* < 0.05) between ABA-free and ABA-added treatments.

The IRT1 activity was previously shown to be tightly regulated by the Fe level supplied to the plant (Vert et al., 2002; Kobayashi and Nishizawa, 2012). Therefore, we first used the Col-0 plants fed with two different levels of Fe to test the correlation between inhibition of IRT1 and decrease of Cd uptake in roots in response to ABA application. In the presence of Cd, the expression of *IRT1* in roots of the plants fed with 5 μM Fe was significantly higher than that of the plant fed 25 μM Fe, but at 0.5 μM ABA concentration IRT1 levels were similar in both (Figure 5A). Similarly, the ACAPCRs of the former plants were also significantly higher than that of the latter plants, but decreased to a similar level by ABA application (Figure 5B). The coordination between the manners of ABA-meditated inhibitions in *IRT1* expression and in Cd uptake supports the assumption that exogenous ABA might decrease Cd uptake via inhibition of IRT1.

**Figure 6:**
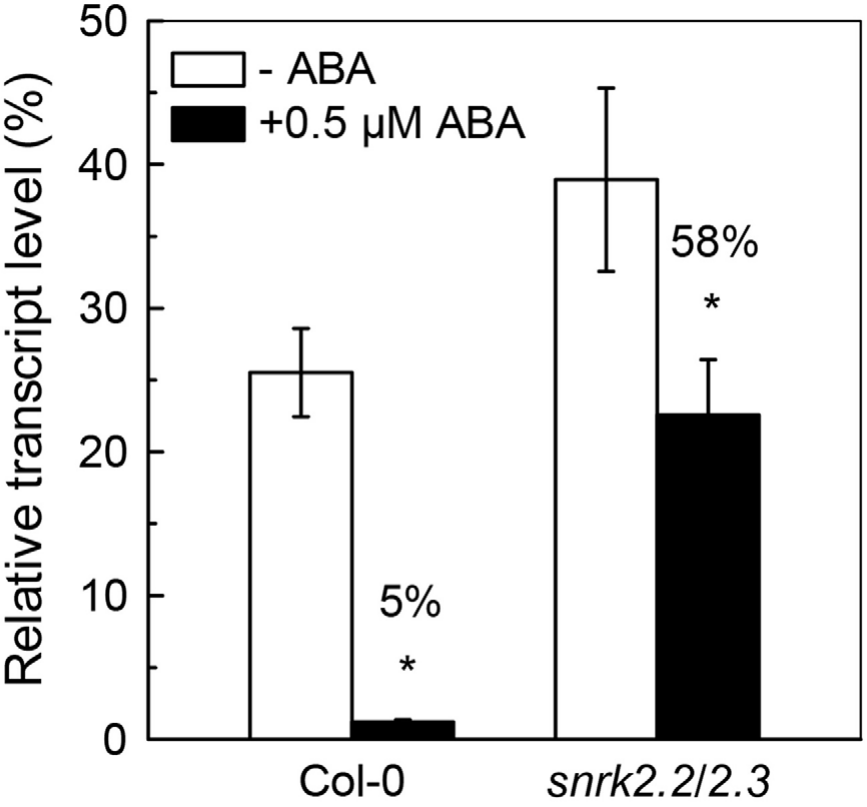
**Effect of ABA on expression of *IRT1* in Col-0 and *snrk2.2/2.3* plants**. Plants were grown in 10 μM Cd-added nutrient solution containing 0 or 0.5 μM ABA for 3 days. The Fe level in the nutrient solution was 25 μM. Transcript level of *IRT1* was normalized to that of *UBQ10* mRNA (100%). Data are means ± SD (*n* = 6). ^*^Significant differences (*P* < 0.05) between ABA-free and ABA-added treatments.

Since the ABA-mediated inhibition of Cd uptake was less in *snrk2.2/2.3* mutant as compared to Col-0 plant (Figure 2C), the effects of ABA application on *IRT1* expressions in roots were compared between these two plant lines. As shown in Figure 6, *IRT1* expressions in Col-0 and *snrk2.2/2.3* were significantly inhibited by 0.5 μM ABA in the presence of Cd. The inhibition was much more predominant in wild-type plants as compared to the mutant plants (Figure 6). This difference in *IRT1* expression correlated with the observation that ABA application resulted in more decrease in ACAPCR in Col-0 plants than in *snrk2.2/2.3* mutant plants (Figure 2C). This correlation further supports the assumption that the decreased Cd uptake resulting from ABA treatment is probably due to the inhibition of IRT1 activity.

**Figure 7:**
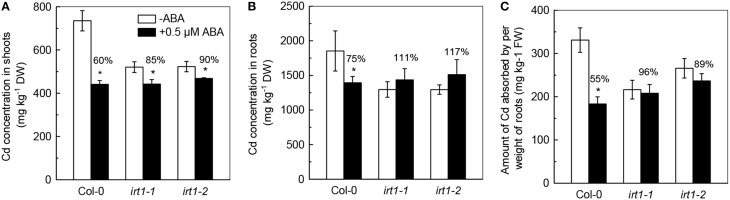
**Effects of ABA on Cd concentration and Cd uptake in Col-0 plants and the *irt1-1*and *irt1-2* mutants**. Plants were grown in 10 μM Cd-added nutrient solution containing 0 (−ABA) or 0.5 μM ABA for 7 days. The Fe level in the nutrient solution was 25 μM. **(A)** Shoot Cd concentrations. **(B)** Root Cd concentrations. **(C)** Amount of Cd absorbed by per weight of roots. Data are means ± SD (*n* = 5). ^*^Significant differences (*P* < 0.05) between ABA-free and ABA-added treatments.

We then investigated the effects of ABA application on Cd levels in *irt1-1*and *irt1-2*, mutants. As shown in Figure 7, the Cd levels in shoots of *irt1-1* and *irt1-2* mutants significantly decreased, whereas the decrease in both mutants was less than that observed in Col-0 plants. In addition, the 0.5 μM ABA application increased the Cd levels in roots of these two *irt1* mutants, although the increase was not statistically significantly, which is in contrast with the results obtained for Col-0 plants. Furthermore, the ACAPCRs in *irt1-1* and *irt1-2* mutants were barely affected by the ABA treatment, whereas a significant decrease of 50% was observed in Col-0 plants. These results verified that ABA-decreased Cd uptake is associated with the inhibition of IRT activity.

## Discussion

This study verified our speculation that the inhibition of root Cd uptake because of ABA pre-treatment (Hsu and Kao, 2003; Uraguchi et al., 2009) may be the result of toxicity by high doses of ABA used for the pre-treatments. We showed that 1 μM of ABA was sufficient to significantly impair the growth of Col-0 plants regardless of Cd treatments. However, application of ABA at the doses less than 1 μM had no significant effect on the growth of Col-0 plants in the absence of Cd. Moreover, this treatment significantly decreased Cd uptake by roots in the presence of Cd, thus decreasing Cd levels in plant organs, alleviating Cd-induced chlorosis in leaves, and improving the growth (Figure 1). These results suggest that inhibition of root Cd uptake by low dose of ABA should be attributed to its signaling function rather its toxic effect. The significant inhibitory effect of ABA on the Cd uptake in Col-0 plants as compared to the *snrk2.2/2.3* mutants, in which the SnRK2.2 and SnRK2.3 mediated ABA signaling pathways are lost (Fujii et al., 2007), further confirmed this observation (Figure 2C).

Various plant hormones are widely used to improve crop productions. For example, auxin and its analogs have been used to increase yield and improve the fiber quality of cotton (*Gossypium barbadense* L.) (Sawan and Sakr, 1998). Gibberellins have been used to delay ripening and reduce berry drop in seedless grapes (*Vitis labrusca* L.) (Bental, 1990). The findings in this present study indicate that exogenous application of low-dose ABA might be also a promising agricultural practice for reducing Cd accumulation in crops grown in Cd contaminated soils. We observed that Fe levels in the growth medium have a distinct impact on the efficiency of ABA induced decrease in Cd uptakes. In the growth medium containing 5 μM Fe, 0.5 μM ABA application resulted in about 55% decrease in the Cd uptake, whereas in the present of 25 μM Fe, the same dose of ABA application decreased the Cd uptake by only about 30% (Figure 5B). Hence, it seems that ABA application might have better efficiency in decreasing Cd contamination in crops grown in calcareous soils where the Fe bioavailability is often severely limited, and which occupy 30% of the earth's surface (Jin et al., 2007; Kim and Guerinot, 2007). It is worth noting that ABA is metabolically unstable due to glycosylation at its carboxylate group and hydroxylation at its cyclohexenone ring (Saito et al., 2004). Accordingly, ABA is easily decomposed by microbes in soil conditions (Hartung et al., 1996). As a result, in field crop production, application of ABA compound into the soils to decrease Cd uptake of crops might be unsuitable. Other alternative techniques need to be developed to avoid this shortfall of ABA application in soils. Interestingly, several fungi such as *Cercospora rosicola, Cercospora cruenta*, and *Botrytis cinerea*, have the abilities to biosynthesize ABA compound (Oritani and Kiyota, 2003). Approaches using these ABA-synthesizing fungi to produce ABA compound *in situ* in rhizosphere soil may be a promising alternative techniques for decreasing Cd contamination in crops. Alternatively, the application of a chemically stable ABA-analog such as AM1 (ABA-mimic 1) may be an alternative. AM1 lacks the carboxylate group as well as the conjugated linker, and thus is chemically more stable than ABA (Cao et al., 2013). Future studies need to be undertaken to clarify the actual effects of the above two proposed techniques on decreasing Cd contamination in crops.

Although it is clear that low-dose ABA application decreases Cd uptake in roots, a question arises about as to how this happens. As mentioned in the earlier sections, IRT1 transporter is not only essential for roots to acquire ferrous iron from soil, but also plays a key role in the Cd uptake (Vert et al., 2002; Lux et al., 2011). Many studies have revealed that modification of root Cd uptake by pharmacological treatment or environment variations associated with the alteration in IRT1 activity. For example, treatment of L-NAME, an inhibitor of nitric oxide syntheses, has been reported to decrease Cd uptake of *Arabidopsis* plants through inhibition of *IRT1* expression in roots (Besson-Bard et al., 2009). Our recent study showed that increase of *IRT1* expression might be a key mechanism involved in nitrate-facilitated Cd uptake in roots of tomato (*Solanum lycopersicum* L.) plants (Luo et al., 2012). In this present study, we found that, in two different Fe-supply growth conditions, the decreases in ACAPCR correlated with the inhibitions of the *IRT1* expression in roots in response to ABA application (Figure 5). Furthermore, the difference in ABA-inhibited *IRT1* expressions between Col-0 and *snrk2.2/2.3* also correlated well with the difference in ABA-decreased ACAPCRs between these two plant lines (Figures 2C, 6). These findings suggested that decrease in ABA-induced Cd uptake may be due to the inhibition of IRT1 activity. This suggestion was further verified by negligible effect on ACAPCR values of IRT1-null mutants, *irt1-1* and *irt1-2* by ABA application (Figure 7).

**Figure 8:**
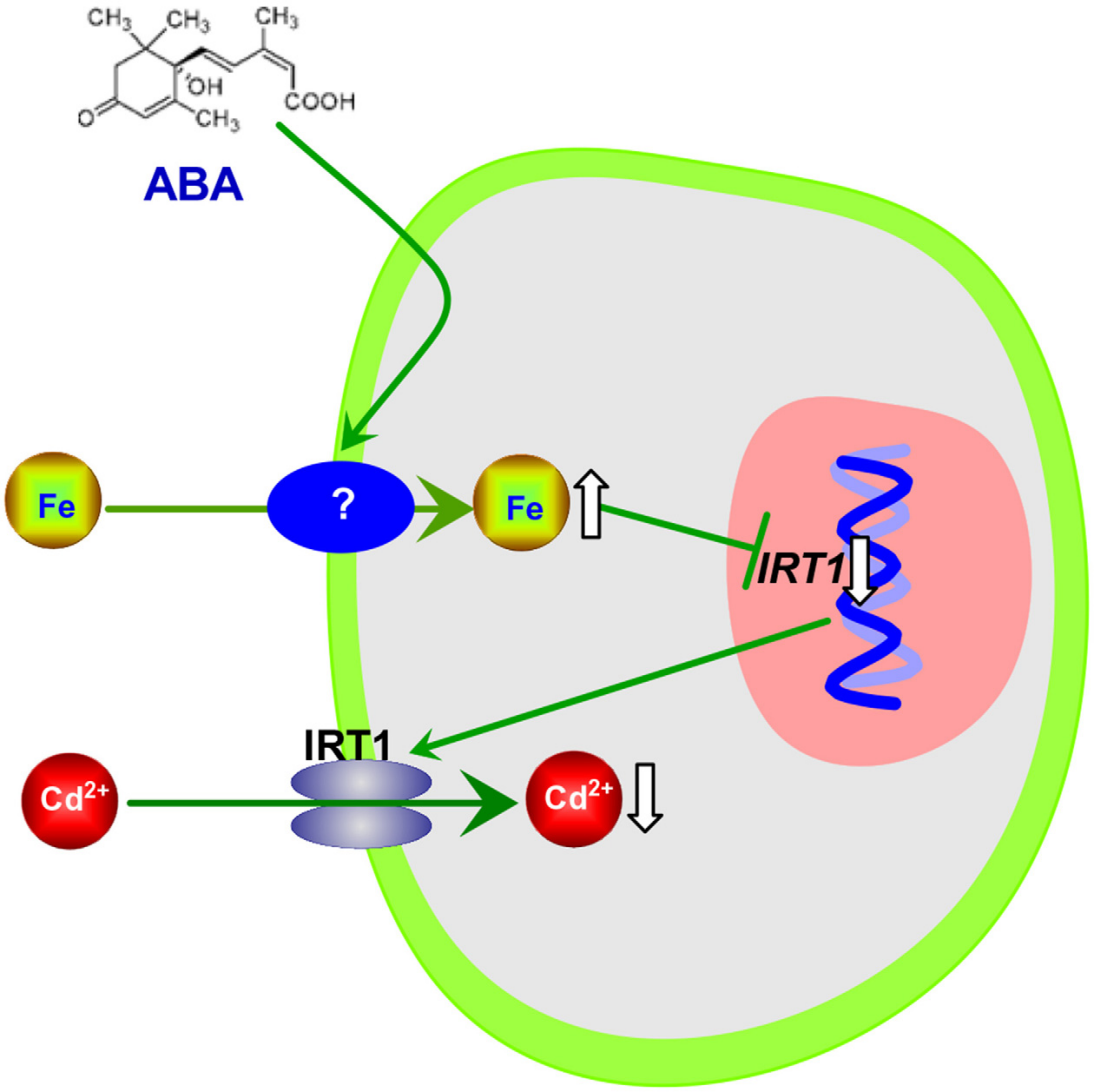
**Schematic model of ABA-mediated decrease in Cd accumulation in *Arabidopsis* plants**.

Interestingly, although IRT1 is the primary Fe transporter (Vert et al., 2002) and ABA application strongly decreased *IRT1* expression in roots of Col-0 plant, exogenous ABA increased Fe concentrations in both Col-0 plants and *irt1* mutants under Cd-exposed conditions (Figures 3, 4). This shows that only IRT1 is not responsible for ABA mediated increase in Fe acquisition. Recently studies have implicated that unidentified plasmalemma pathway may also play a role in iron acquisition by roots (Jin et al., 2014). Iron levels in red clover and *Arabidopsis* fed with Fe (III)–siderophore which is barely reduced to Fe^2+^ by ferric chelate reductase for the subsequent uptake by IRT1, were found to be higher than in those fed with Fe–EDTA (Vansuyt et al., 2007; Jin et al., 2010). Another question arises about the mechanism(s) involved in inhibition of *IRT1* expression in response to exogenous ABA. It is well-known that the expression of *IRT1* in roots is negatively regulated by the Fe status in plants (Connolly et al., 2002; Vert et al., 2002; Kobayashi and Nishizawa, 2012). We propose the following model in which exogenous ABA application results in IRT1-independent Fe acquisition, by an unidentified plasmalemma pathway and consequent elevation of iron level in plants resulting in inhibition of *IRT1* expression in roots. As a result, the IRT1-mediated Cd uptake by roots is decreased, and plant growth is improved (Figure 8).

For crop production in Cd contaminated soil, developing techniques based on ABA application (e.g., *in situ* synthesis of ABA by microorganisms and application of stable ABA-analogs in soil) might provide a promising approach to reducing Cd accumulation in edible organs, thus improving food safety. It is worth mentioning that ABA application also significantly decreases the Cd translocation from roots to shoots in Col-0 plants (Supplementary Figure 1). Since Cd is highly toxic to plants, decrease in Cd translocation into shoots was proposed to protect important organs of shoots (Gong et al., 2003; Li et al., 2010). Therefore, the inhibition of Cd translocation from roots to shoots by ABA may also contribute to Cd tolerance. Nevertheless, we also observed that inhibitory effect of ABA on Cd translocation in Col-0 plants and *irt1* mutants were very similar (Supplementary Figure 2), indicating that inhibition of Cd translocation by ABA is independent of IRT1. The mechanism of ABA-mediated inhibition in Cd translocation remains unclear, and further studies are necessary to reveal this.

### Conflict of interest statement

The authors declare that the research was conducted in the absence of any commercial or financial relationships that could be construed as a potential conflict of interest.
