# Supplementary material for: Exogenous abscisic acid application decreases cadmium accumulation in Arabidopsis plants, which is associated with the inhibition of IRT1-mediated cadmium uptake
Source: Front Plant Sci. 2014 Dec 16;5:721. doi: 10.3389/fpls.2014.00721 (PMC4267193; doi:10.3389/fpls.2014.00721)
Supplement: Supplementary file 1 [file DataSheet1.DOC]

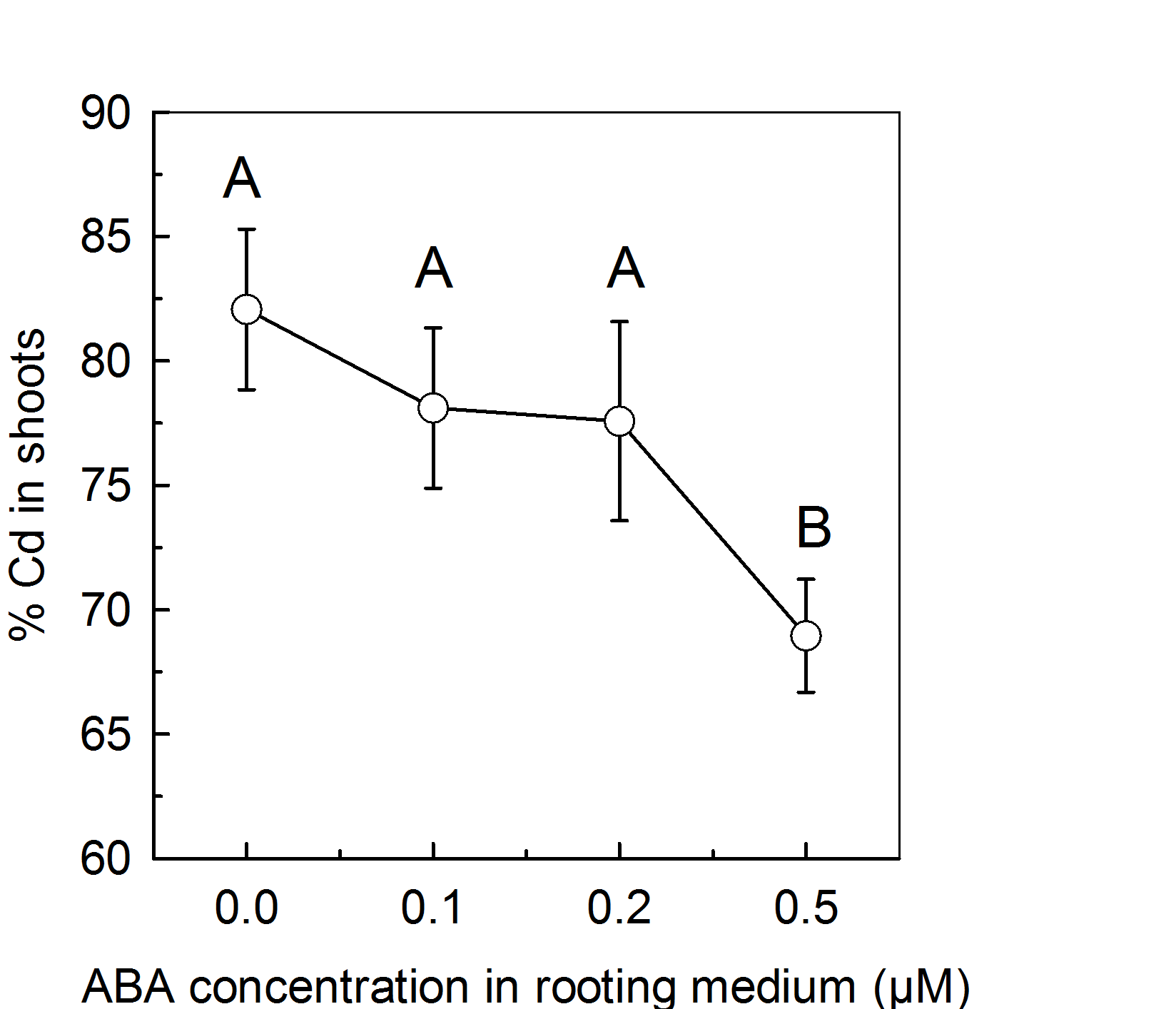


**Supplementary Figure 1. Effects of varying doses of ABA on Cd translocation of Col-0 plants.** Treatments are the same as in Figure 2. Data are means ±SD (n=5). Different letters represent significantly different values at *P<0.05*.


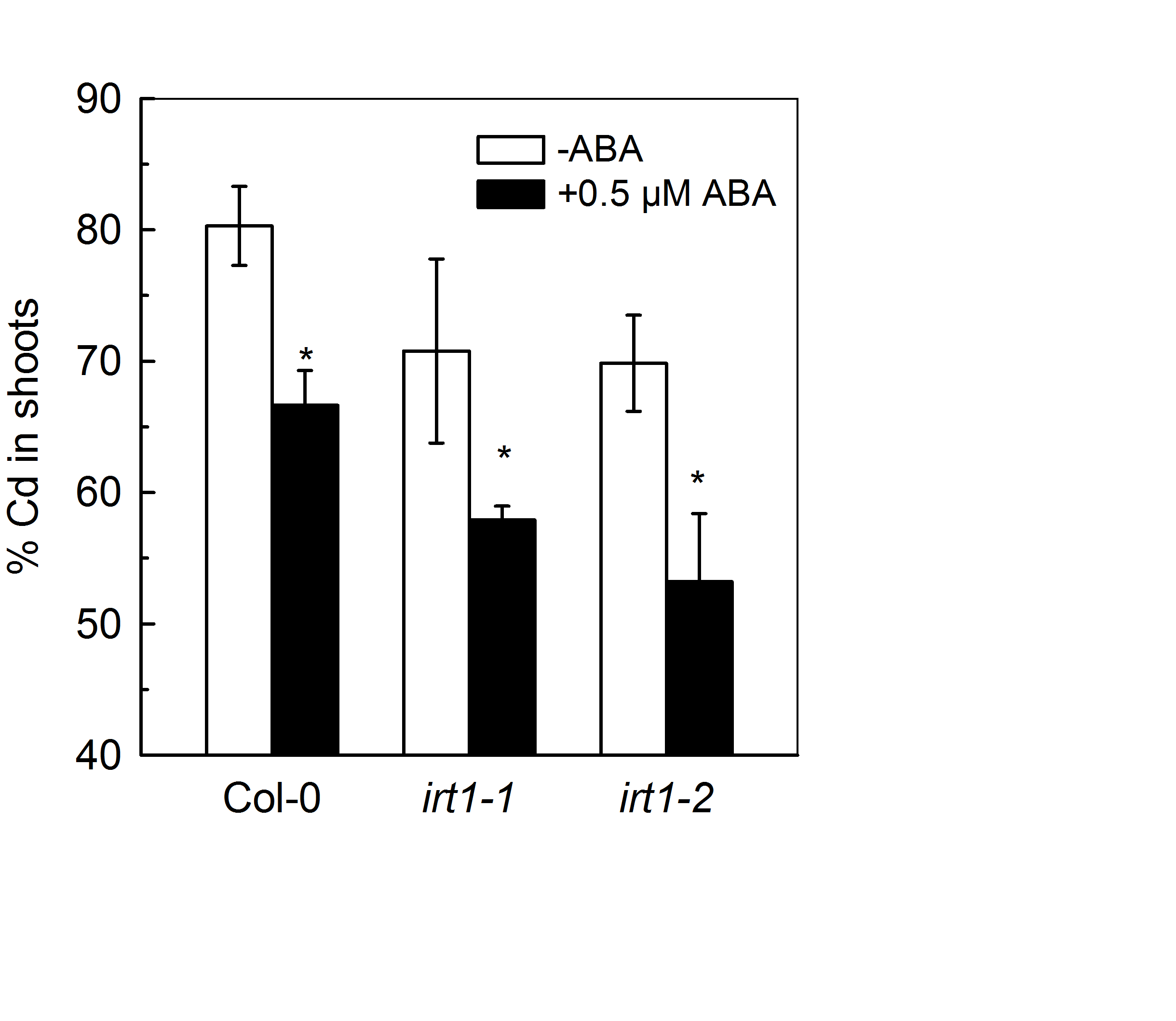


**Supplementary Figure 2. Effect of ABA on Cd translocation in Col-0 plants and the *irt1-1* and *irt1-2* mutants.** Treatments are the same as in Figure 4. Data are means ±SD (n=5). *Significant differences (*P*<0.05) between ABA-free and ABA-added treatments.
